# Wolf Predation Likely Eliminated a Beaver Colony in a Drought‐Stressed Pond

**DOI:** 10.1002/ece3.74472

**Published:** 2026-10-21

**Authors:** Tom A. Diserens, Marcin Brzeziński, Marcin Churski, Dries P. J. Kuijper, Andrzej Zalewski

**Affiliations:** ^1^ Mammal Research Institute Polish Academy of Sciences Białowieża Poland; ^2^ Faculty of Biology University of Warsaw Warszawa Poland

**Keywords:** beaver, hydrological drought, landscape of fear, predation, risk allocation, wolf

## Abstract

Wolves and beavers increasingly co‐occur across the Northern Hemisphere, yet direct observations of their interactions remain rare, and little is known about how beavers respond behaviourally to predation risk under natural conditions. On 29 December 2024, a wolf killed an adult beaver on a foraging trail in Białowieża Forest, Poland, among the first video‐documented wolf–beaver predation events in Europe. Over the 53‐day monitoring period prior to the predation event, wolves visited the trail on 4 days, while beavers were active on 33 of those days (62%), averaging 4.9 detections per day (7.9 on days with activity). This contrasts with experimental work showing that predator cues alone are often enough to sharply suppress beaver activity. The predation occurred during hydrological drought. The watercourse above and below the pond was nearly dry, likely leaving the colony with nowhere safer to go. We interpret this apparent risk tolerance as consistent with the risk allocation hypothesis, where prey with no low‐risk foraging options cannot sustain antipredator behaviour without compromising essential foraging. Following the predation, the pond remained abandoned for at least 18 months. This expands on a previous report of ponds becoming inactive following wolves predating on dispersing beavers, suggesting predation can also displace established colonies, with potentially cascading consequences for water retention, nutrient cycling and vegetation dynamics. In drought‐affected landscapes, wolf predation could plausibly act as a spatial filter, disproportionately removing these ecosystem engineers from suboptimal habitats. This is a hypothesis that warrants future testing. As droughts intensify under climate change, this could compound into a feedback loop. Fewer active beaver sites would mean less landscape‐scale water retention, deepening drought and further constraining the beaver habitat that remains, and undermining the ecosystem functions that make beaver recolonisation so ecologically valuable.

## Graphical abstract

We document a rare video recording of a wolf killing an adult beaver on a foraging trail in Białowieża Forest, Poland, preceded by weeks of beaver activity on the same trail despite repeated wolf visits, and followed by complete pond abandonment lasting at least 18 months. The predation occurred during hydrological drought, when the watercourse above and below the pond was nearly dry, likely preventing the colony from relocating to safer habitat. We interpret the observed risk tolerance as consistent with the risk allocation hypothesis, and discuss the implications of wolf predation for beaver‐driven ecosystem engineering under increasingly frequent drought conditions.

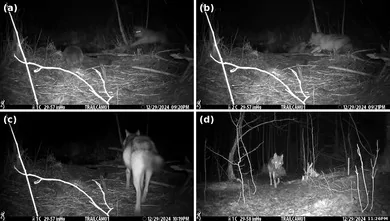

## 1 Introduction

Despite their increasing co‐occurrence across the northern hemisphere, we have at best only a partial picture of how wolves ( *Canis lupus* ) and beavers (*Castor* spp.) interact with one another. Predation and diet studies have shown wolves regularly kill (Gable, Windels, et al. 2018; Gable and Windels 2018) and eat beavers (Mysłajek et al. 2021; Nowak et al. 2024; Sidorovich et al. 2017). Yet observations of wolves hunting beavers are rare (Freund et al. 2025; Gable, Stanger, et al. 2018), leaving open which behavioural patterns and microhabitat conditions make beavers vulnerable to predation, and what wider indirect effects wolves have on beavers, including any trophic cascades these interactions may set off (but see Gable et al. 2020). As both species are often heralded as saviours of biodiversity (Mech 2012; Stringer and Gaywood 2016), this knowledge is urgently needed to predict the ecological effects of the ongoing wolf and beaver recolonisations of Europe and North America.

Prey living alongside predators navigate a ‘landscape of fear’, in which predation risk varies across space and time (Gaynor et al. 2019; Laundré et al. 2001; Palmer et al. 2022). To reduce their likelihood of being killed or disturbed, prey can alter behaviours such as habitat use, temporal niches and vigilance (Gaynor et al. 2019). Such ‘nonconsumptive’ effects have been observed in various prey and mesocarnivore species (e.g., Bubnicki et al. 2019; Prugh et al. 2023), and can lead to prey and their ecological impacts being absent from parts of the landscape they perceive to be risky (Gaynor et al. 2019). Yet it remains poorly understood how these dynamics play out for semi‐aquatic prey such as beavers, whose access to refuge is tied to water rather than landscape‐scale movement.

Prey responses to risk depend on the ecological traits of predator and prey, and the context they inhabit (Haswell et al. 2017; Kuijper et al. 2016). Beavers are fossorial, semi‐aquatic mammals that rely on water and structural refuges—lodges or riverbank burrows—to avoid predators, but they must often risk emerging onto land to forage when aquatic vegetation is limited, particularly in winter. To make this trade‐off effectively and maximise their own fitness, they need to assess how risk varies in space and time and adapt their terrestrial movements accordingly. The ultimate behavioural response is based on a cost–benefit calculation weighing up risk avoidance against other critical life functions, such as food acquisition or mating (Diserens et al. 2022; Gaynor et al. 2019). Experimental studies have demonstrated that beavers avoid foraging trails and food with predator cues on them (Rosell and Czech 2000; Salandre et al. 2017; Severud et al. 2011), but it remains unclear whether beavers sustain these behavioural responses in the wild (Parsons et al. 2018). If they do, this could have cascading effects on the many ecological functions beavers perform as ecosystem engineers (Stringer and Gaywood 2016).

Beavers' semi‐aquatic lifestyle likely shapes their response to wolf predation risk in ways not captured by better‐studied wolf–ungulate systems (Ripple et al. 2014). Unlike ungulates, which can move relatively unpredictably across the landscape to avoid risk, beavers are spatially constrained to the vicinity of water and their lodges or burrows. They also cannot easily relocate to safer areas without risking intraspecific aggression (De Stefano et al. 2006; Mayer et al. 2020). This creates a binary risk landscape, with low risk in water or refuge and high risk on land. Foraging is also highly predictable in space, concentrated within roughly 500 m of the lodge or burrow (Nolet et al. 1994). For mobile prey, avoiding risk and finding food can often be reconciled by simply moving elsewhere; for beavers, the two are settled by a single binary choice—stay in refuge or accept elevated risk to forage. When no safer options are available, they may have no choice but to tolerate that risk (Gaynor et al. 2019).

Here, we document a rare video recording of a wolf killing an adult beaver ( *Castor fiber* ) in Białowieża Forest, Poland, preceded by a counterintuitive pattern of risk tolerance—beavers continued foraging on a single trail despite repeated wolf visits in the weeks before the kill—followed by the elimination of this keystone ecosystem engineer from the site. This observation prompted us to (1) describe the predation event and the beaver's and wolf's behaviour; (2) document beaver activity and predator visits at the pond before and after the kill, to explore whether beavers altered their behaviour in response to wolf visits or the kill; and (3) review the literature on beaver responses to predation risk and identify directions for future research.

## 2 Methods

### 2.1 Study Area

The study took place in Białowieża Forest, one of the best‐preserved woodlands in the European lowlands. While tree stands in the forest are relatively well‐preserved, its river valleys exhibit high landscape heterogeneity: rivers are flanked by mowed meadows, natural wet meadows, old‐growth tree stands, natural secondary growth or forestry plantations. The focal river, the Łutownia, a left tributary of the Narewka with a catchment area of approximately 121 km^2^, is a small river (1–5 m wide, < 1 m deep) characterised by low gradients and shallow channels, and was historically regulated, flowing down a straightened channel at the centre of its valley.

The winter preceding the predation event was dry. Monthly precipitation in Hajnówka (Polish national meteorological service, ~8 km from the study site) was below the 1991–2020 normal in nine of the 12 months of 2024, with October–December particularly deficient (Figure 1); annual precipitation totalled 566 mm, against a long‐term normal of 623 mm. Hydrological drought is a recurrent and worsening feature of Białowieża Forest, and the Łutownia is particularly vulnerable to low flows, with reaches periodically running dry in summer (Grygoruk et al. 2022; Hejduk et al. 2021), consistent with widespread observations of dry riverbeds and abandoned beaver territories across the forest in recent years (T.A. Diserens, personal observation).

**FIGURE 1:**
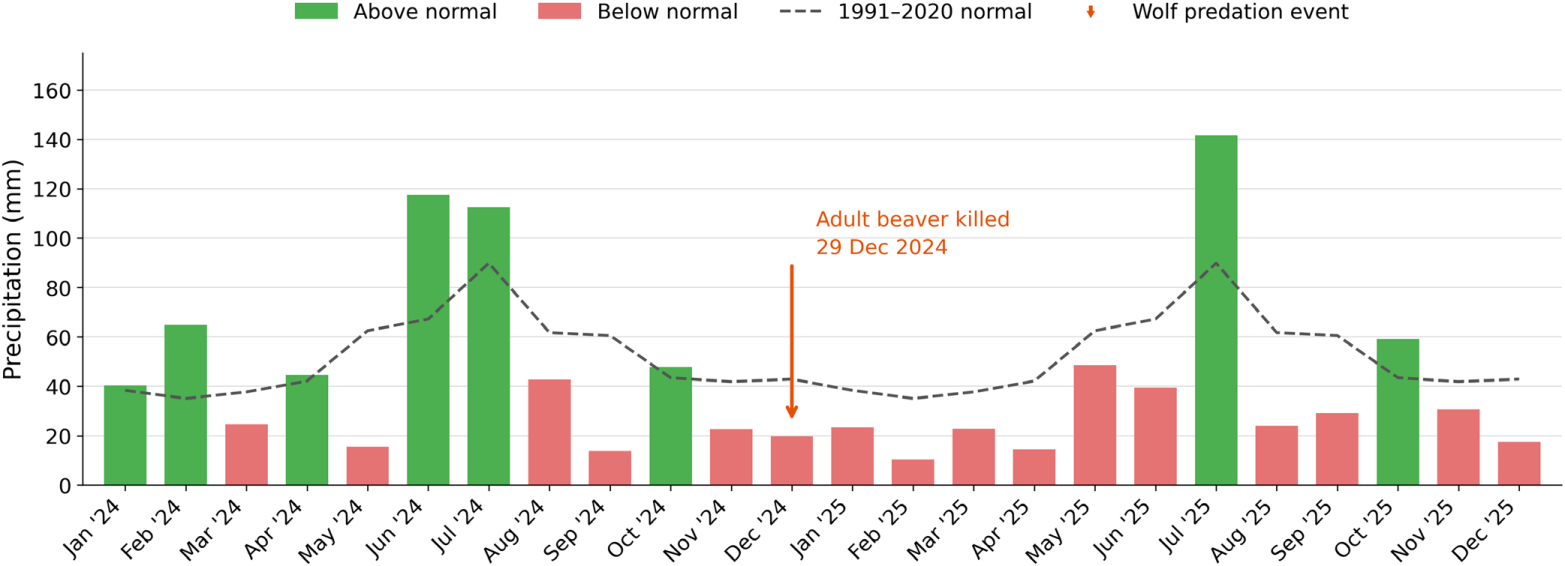
Monthly precipitation at Hajnówka meteorological station (IMGW‐PIB, Poland; ~8 km from the study site), January 2024–December 2025. Bars indicate monthly totals, coloured by departure from the 1991–2020 normal (dashed line). The arrow marks the wolf predation event on an adult beaver on 29 December 2024. Precipitation is shown to illustrate the drought conditions preceding the predation event, discussed as a contributing factor to reduced water levels and beaver vulnerability.

Beavers recolonised the Polish side of the forest in the 1980s (personal comm. E. Bujko), following extirpation and subsequent legal protection in 1919, and subsequently expanded into smaller and more marginal watercourses, including seasonal watercourses, drainage ditches and bogs with only seasonal standing water. In recent years, however, worsening drought has led beavers to abandon many of these marginal sites (T.A. Diserens, personal observation). Beavers co‐occur in the forest with wolves (their main predator) and lynx ( *Lynx lynx* ), both strictly protected.

The study beaver pond (50–100 m^2^, Figure 2) was created by a dam approximately 30 m upstream of a forest road bridge and embankment, and sits within a 160 m wide, low‐gradient floodplain of old‐growth black alder ( *Alnus glutinosa* ) bog forest. Between the pond's edge and the wooded foraging area beyond, a largely treeless strip of land up to 30 m wide meant beavers had to cross exposed ground to reach food. Beaver foraging activity was concentrated on a single 50‐m trail opposite the lodge, cutting through a hazel ( *Corylus avellana* ) understory beneath the alder canopy, with few other cut stems within 100–200 m of the dam. Upstream, water levels dropped below 20 cm ~150 m from the dam, and the river was largely dry for the next 4 km to the nearest beaver territory; downstream, water was similarly shallow (~10 cm) for ~200 m before a separate colony's cascade of at least six dams. This hydrological isolation severely limited the colony's options for relocating to better‐watered habitat. We conducted follow‐up terrestrial surveys of the area extending 50 m upstream and downstream of the dam in July, August and December 2025, and July 2026 searching for signs of recent beaver activity (fresh cut stems, trails or other feeding signs).

**FIGURE 2:**
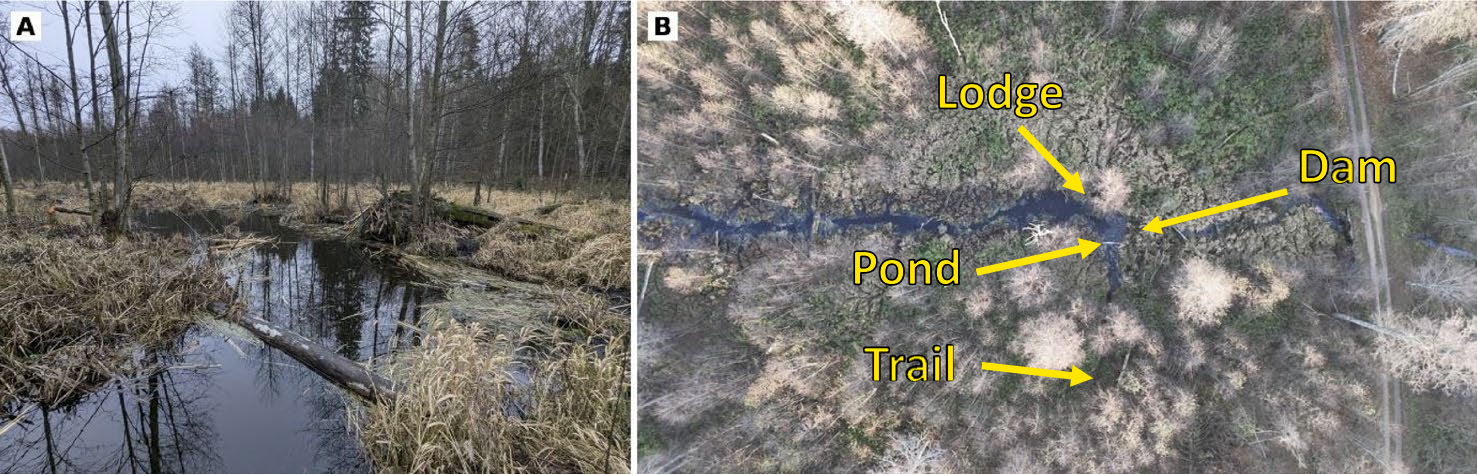
The beaver pond and its surroundings. (A) Photograph of the area behind the beaver impoundment; the lodge is visible in the centre right, the dam lies out of frame to the right, and the foraging trail runs inland from the bottom left. (B) Drone image of the pond taken on 6 November 2024, with the pond, lodge, dam and main foraging trail visible. The trail extended approximately 55 m from the water's edge; cameras were deployed along it at 10, 25, 45 and 55 m, with a further camera at the lodge.

### 2.2 Camera Trapping and Video Processing

On 6 November 2024, we deployed camera traps on the colony's main foraging trail, which was directly opposite the lodge, and at the lodge itself. Cameras were programmed to record 10‐s videos when triggered by motion. We initially deployed three cameras: one at the lodge and two on the foraging trail facing the water, at approximately 10 and 25 m from the water's edge, using LTL Acorn SGN‐5310 cameras. On 10 December 2024 these were replaced with Browning Dark Ops Pro X 1080 cameras at the same positions. From 6 February 2025 the lodge camera was removed and two additional cameras were deployed further along the trail at approximately 45 and 55 m from the water's edge, together providing continuous coverage of the trail's full length. This approach was aimed at recording beaver and wolf behaviour at varying distances from water along beaver trails. Cameras were checked every 2–8 weeks to replace batteries and SD cards. A full deployment timeline is shown in Figure A1. All cameras were removed on 20 March 2025 after approximately 3 months without beaver detections.

Videos were uploaded to Trapper, a web‐based platform for camera trap data management (Bubnicki et al. 2016), where we classified the species and number of individuals present in each video. We quantified beaver activity as the number of distinct 5‐min intervals per day in which beavers were detected, hereafter ‘detections’. Daily activity and predator visit data were plotted in Python (version 3.12; Python Software Foundation) using pandas and matplotlib.

## 3 Results

On 29 December 2024 at 21:20, a wolf attacked an adult beaver on land, a few metres from the water's edge, along the colony's primary foraging trail (Video 1). Earlier that day, beaver activity totalled 46 detections. The camera was triggered as the beaver fled down the foraging trail toward the river. The wolf intercepted it by approaching perpendicular to the trail, grabbed it by the neck and dragged it away from the water. During the struggle, the beaver briefly escaped on two occasions, but the wolf recaptured it—first by biting its side, then its leg—before pulling it out of the camera's frame to the right. The moment of killing occurred outside the camera's view.

**VIDEO 1:** Camera‐trap footage documenting the wolf predation event on a Eurasian beaver on 29 December 2024 in Białowieża Forest, Poland. This video is a compilation of individual motion‐triggered camera‐trap clips, joined together with no other editing performed; clip length varied due to a combination of low battery and cold weather causing the cameras to shorten recording duration. Footage was recorded on the primary foraging trail of a beaver colony on the Łutownia River. The sequence shows typical beaver activity on the trail at 21:13, the wolf attack at 21:20, the wolf walking up the trail at 21:37 and the wolf carrying the beaver carcass at 22:19. All footage is from the trail camera 10 m from the water's edge, except the final clip, recorded on the trail camera 25 m from the water's edge. The timestamp displayed in the final clip reads 07:02 and was not corrected in the video; the true time of recording was approximately 23:26. Video content can be viewed at https://onlinelibrary.wiley.com/doi/10.1002/ece3.74472.

Later, at 21:37, a wolf walked past the camera from the right. At 22:19, a wolf walked past the camera from the left with a beaver carcass in its mouth, with the beaver's tail clearly visible. At 23:26, a wolf was recorded on a separate camera positioned further from the water on the same foraging trail, carrying what appeared to be the beaver pelt in its mouth. Figure 3 shows four still frames from the evening of the predation event.

**FIGURE 3:**
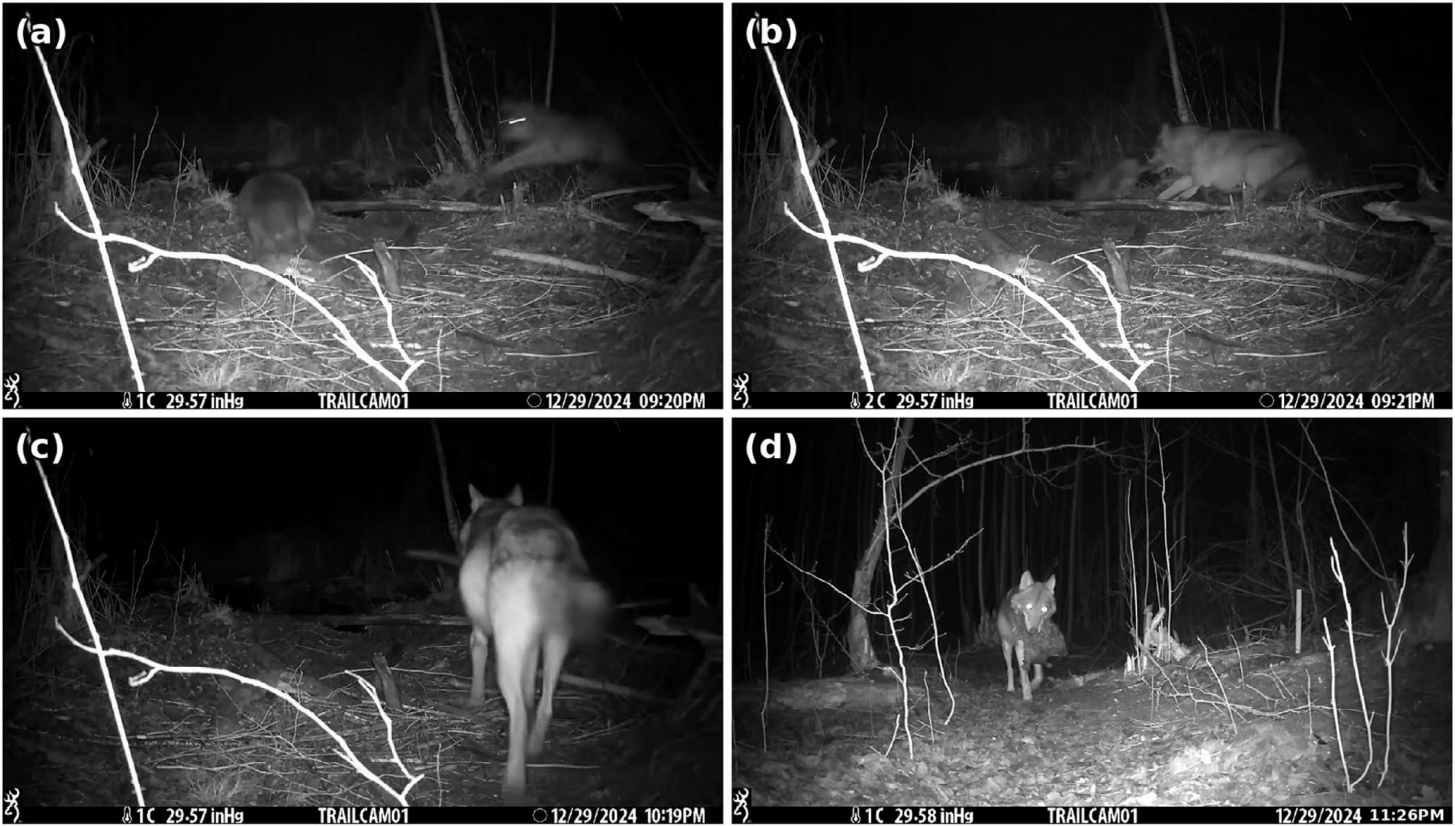
Still frames from camera‐trap videos documenting the wolf predation event on a Eurasian beaver on 29 December 2024 in Białowieża Forest, Poland. (a) At 21:20, the wolf pounces from the right as the beaver, apparently alerted to the predator's presence, flees toward the water. (b) At 21:21, the wolf drags the beaver away from the water by the leg. (c) At 22:19, the wolf carries a beaver carcass, with the beaver's tail visible. (d) At 23:26, recorded on a separate camera positioned further from the water on the same foraging trail, the wolf is seen carrying what appears to be the beaver pelt in its mouth.

Prior to the predation event, beavers were frequently recorded on the foraging trail, despite repeated wolf visits (Figure 4). Over this 53‐day period, wolves visited the trail on 4 days, rising to 5 once the camera at the lodge was included; one of the trail visits, on 10 December 2024, involved a group of four wolves. Beavers were active on 33 of the 53 days (62%), averaging 4.9 detections per day (7.9 on days with activity). Camera trap videos captured two beavers simultaneously on several occasions, and on at least one occasion a beaver was recorded fleeing toward the water with no wolf visible in the same video.

**FIGURE 4:**
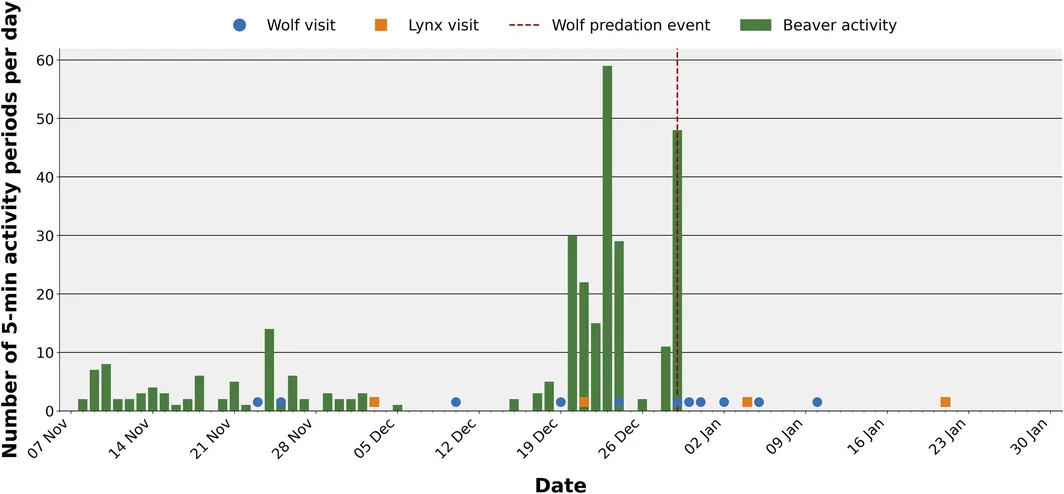
Temporal patterns of beaver activity and large predator visits at the beaver foraging trail in Białowieża Forest, Poland, showing beaver activity before and after a wolf predation event on 29 December 2024. Bars show daily beaver activity, quantified as the number of distinct 5‐min activity periods per day derived from camera‐trap videos, which controls for variation in video length and trigger frequency across the deployment; activity is restricted to the trail camera 10 m from the water's edge, the only camera deployed throughout the study period. Points indicate days on which wolves and lynx were recorded, pooled across all cameras. The wolf predation event is marked by a dashed vertical line. The figure illustrates that beavers continued using the trail despite repeated wolf visits in the weeks prior to the predation event, and that no beaver activity was detected after it. The *x*‐axis is restricted to end of January 2025 for clarity; subsequent wolf visits are described in the text.

In the week following the predation event, wolves visited on three separate days, including a visit by two wolves on 2 January 2025. Over the subsequent 2.5 months they were observed on three further days, including 5th and 6th March 2025—beyond the range shown in Figure 4. Lynx were recorded on the trail in two detections before and two after the predation event. No beaver activity was detected on any camera for the remainder of the monitoring period, which ran until 20 March 2025, and terrestrial surveys in July, August and December 2025, and July 2026 found no fresh signs of beaver activity within 200 m of the dam.

## 4 Discussion

Our observations provide a rare insight into beaver responses to wolf risk and predation, and their potential ecological consequences. We report a video of a wolf preying on an adult beaver, preceded by frequent beaver use of a foraging trail that was repeatedly visited by wolves, and followed by the elimination of the colony. By capturing both the events leading up to and following predation, this study expands on previous reports of wolf–beaver predation events (Freund et al. 2025; Gable, Stanger, et al. 2018). The event occurred during a period of drought, when the watercourse above and below the pond was nearly dry. We suggest later in the discussion how these constrained habitat conditions may have limited the beaver's ability to mitigate risk. We present possible explanations for the observed risk tolerance, explore how beavers navigate landscapes of fear, and identify priorities for future research.

The behaviour of the predating wolf was similar to the two previously published recordings of wolves hunting beavers (Freund et al. 2025; Gable, Stanger, et al. 2018). As in those videos, the wolf repeatedly attacked and bit the beaver from behind, dragging it away from water, seemingly taking care to protect itself from being bitten by intermittently releasing its prey. We agree with the interpretation of Freund et al. (2025) that the apparent dragging of the beaver away from the water suggests wolves recognise that beavers are more vulnerable on land. A novel aspect of our observation is the wolf preventing the beaver from reaching the water by grasping its foot, a behaviour that, to our knowledge, has not been previously documented. The wolf's direction of approach, perpendicular to the foraging trail, suggests it was moving along the river shoreline and encountered the beaver opportunistically, consistent with a cursorial rather than a sit‐and‐wait ambush strategy (Gable et al. 2016). Both, dragging beavers away from water and opportunistic hunting have been documented elsewhere (Gable, Windels, et al. 2018), suggesting these are broadly employed strategies. On two occasions, more than one wolf was recorded together in a clip, suggesting beaver hunting here was not the behaviour of a single specialised individual. Wolves also kept visiting for months afterward—well beyond any plausible window for residual carcass scent—but camera‐trap footage cannot distinguish individuals, so whether this reflects continued hunting effort at a known site or just routine patrolling of the trail cannot be resolved with the available data.

Contrary to experimental studies that showed beavers avoid trails and food with experimentally added predator cues (Rosell and Czech 2000; Salandre et al. 2017; Severud et al. 2011), we observed beavers repeatedly using the same foraging trail despite recurring visits of actual wolves (and lynx). The repeated predator visits suggest wolves and lynx were attracted to the trail by olfactory cues left by the beavers (Parsons et al. 2018). This raises the question of what causes beavers to tolerate such risk under natural conditions. Predator–prey theory offers a possible explanation. The risk allocation hypothesis (Lima and Bednekoff 1999) predicts that when high‐risk periods are frequent or prolonged, prey have little choice but to continue foraging despite elevated risk, as the energetic cost of sustained antipredator behaviour becomes incompatible with survival.

In this case, habitat quality was clearly suboptimal. Optimal water depths for beavers are typically 0.6–1.0 m (Rosell and Campbell‐Palmer 2022), yet upstream of the dam only a 150 m stretch of river reached this depth. Beavers typically forage across multiple trails, which reduces their spatial predictability to predators; here, foraging was concentrated on a single, long trail—likely a consequence of the severely constrained habitat. Foraging further from water increases predation risk (Gable et al. 2023), and low water levels compounded this by shrinking the aquatic refuge available near the trail. Higher water levels would also have given the colony more forage within safe reach of water. With no safer alternative, the colony had little choice but to keep using the trail despite repeated wolf presence—precisely the condition under which the risk allocation hypothesis predicts a moderated rather than avoidant response to risk.

Previous studies of the wolf diet provide broader context for this interpretation. A study in Belarus documented that in dry years beavers as a proportion of biomass in the wolf diet rose sharply, up to almost 50%, compared to below 20% in wetter years (Sidorovich et al. 2017). Similarly, wolves in northeast Poland consumed far more beavers during drier years, with beaver biomass in the wolf diet rising from 11% in the wettest year to 41% in the driest (Mysłajek et al. 2021). These patterns are consistent with the idea that drought increases beaver vulnerability by reducing aquatic refuge and forcing longer‐distance terrestrial foraging (Gable et al. 2023; Gable, Windels, et al. 2018). However, we cannot discount that drought increases beaver mortality via some other mechanism—such as reduced body condition—and that wolves are simply scavenging or opportunistically killing beavers that are already more vulnerable for reasons unrelated to their behaviour on land.

Our observation of the pond remaining vacant for at least 18 months after the wolf predation event goes beyond that of Freund et al. (2025), who reported a 96% decline in beaver activity on a foraging trail following a predation event. The fate of the second adult beaver remains unknown; it may have been predated, died of other causes or relocated. Whether any additional individuals, such as kits or yearlings, were present at the pond could not be determined from the available footage. While Gable et al. (2020) found ponds became inactive once wolves killed the dispersing beaver that had colonised them, our observation suggests that wolf predation can also eliminate small established colonies. If wolf‐induced pond abandonment occurs with any regularity and persists beyond short time frames, such events could generate localised cascading effects (Johnson‐Bice et al. 2023) in landscapes where wolves and beavers coexist. Through direct or indirect pathways, wolves may exclude beavers from parts of the landscape, potentially altering local water retention, nutrient cycling, vegetation structure and forest succession. In areas such as Białowieża Forest where rivers were historically regulated by people, wolf predation may also reduce beavers' capacity to renaturalise waterways (Larsen et al. 2021). The dam itself remained intact throughout the period of abandonment, but had little effect on water levels given the ongoing drought. As of summer 2026, the pond remained abandoned; longer‐term consequences will depend on how long abandonment persists, and on whether rainfall recovers enough for the site to become viable again.

The return of wolves and beavers across much of the Northern Hemisphere has fuelled debate over their ecological roles and management implications (Halley and Rosell 2002; Kuijper et al. 2019, 2024; Larsen et al. 2021). Both are keystone species whose interactions are likely to shape ecosystem responses to nature restoration and climate change. Our observation provides rare insight into the behavioural and ecological consequences of wolf predation and potential predation risk for beavers. The apparent lack of antipredator response prior to predation, followed by elimination of the colony, is consistent with existing theory on landscapes of fear and risk allocation (Gaynor et al. 2019; Lima and Bednekoff 1999), and with descriptive studies showing a higher proportion of beavers in the wolf diet during drought (Mysłajek et al. 2021; Sidorovich et al. 2017).

We think it is plausible that wolf predation could act as a spatial filter, disproportionately removing beavers from marginal habitats where behavioural responses to risk are constrained—a hypothesis that should be tested in future studies. If this holds, its consequences could compound under climate change. More frequent droughts would restrict beaver behavioural responses to predation risk, leaving more sites vulnerable to wolf‐driven abandonment. Fewer active beaver sites would mean less landscape‐scale water retention. This in turn would deepen drought and further constrain the beaver habitat that remains—a feedback loop worth attention in both ecological research and conservation planning. Two related testable questions arise. First, how do beaver behavioural responses to predation risk vary with habitat quality, particularly water availability and the degree of intraspecific competition? Second, under what conditions does wolf predation trigger pond abandonment, and how long does that abandonment tend to persist? Answering these will help guide conservation and management, including conflict mitigation, as wolves and beavers continue to recolonise their former ranges.

## Author Contributions

**Tom A. Diserens:** conceptualization (lead), funding acquisition (lead), visualization (lead), writing – original draft (lead), writing – review and editing (lead). **Marcin Brzeziński:** conceptualization (supporting), funding acquisition (supporting), writing – review and editing (supporting). **Marcin Churski:** conceptualization (supporting), funding acquisition (supporting), writing – review and editing (supporting). **Dries P. J. Kuijper:** conceptualization (supporting), funding acquisition (supporting), writing – review and editing (supporting). **Andrzej Zalewski:** conceptualization (supporting), funding acquisition (supporting), writing – review and editing (supporting).

## Funding

This work was supported by Narodowe Centrum Nauki (2024/52/C/NZ8/00332 and 2021/41/B/NZ8/00015).

## Conflicts of Interest

The authors declare no conflicts of interest.

## Figures and Tables

**FIGURE A1:**
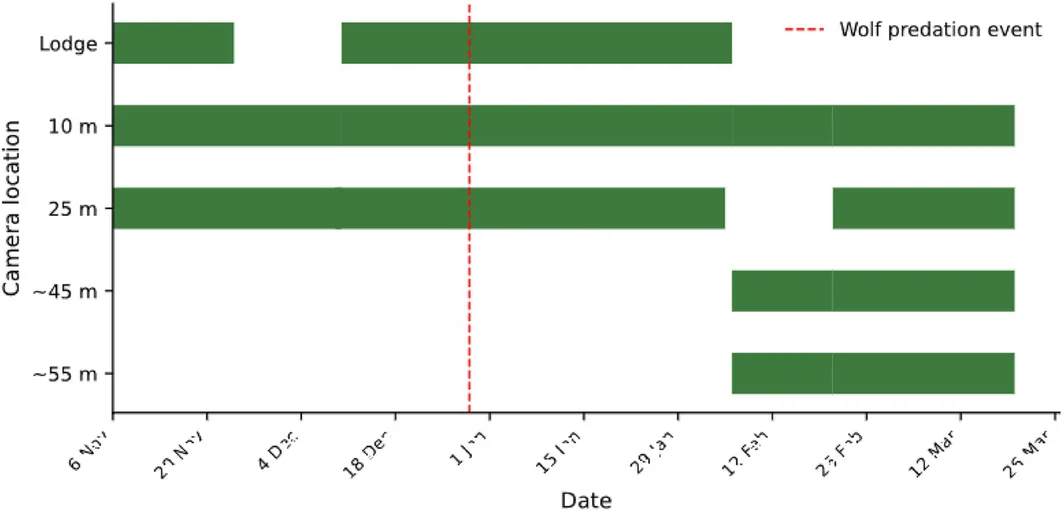
Camera trap deployment timeline at the study pond, 6 November 2024–20 March 2025. Cameras were positioned at the lodge and at approximately 10, 25, 45 and 55 m along the main foraging trail from the water's edge. The dashed red line marks the wolf predation event on 29 December 2024.

## Data Availability

No datasets were generated or analysed. All relevant data, such as video material and photos, are included in the paper.
